# Annotations

**Published:** 1900-01-13

**Authors:** 


### ANNOTATIONS.

### Medical Organisation.

THE curious attempt which is being made to organise the medical profession for the promotion of all kinds of high ideals bv the institution of a medical guild does not seem tc nake much progress; and, indeed, we can hardly wonder at it. The strong feeling which permeates a large number of the most hardworking members of our profession against the whole system of provident dispensaries as at present conducted stands as an absolute bar to any general co-operation between those who support and encourage that system and those who oppose it. The fact is that the profession is being disintegrated by the hard personal pressure of what is in many cases a degrading downward competition, which, although at first no doubt merely a competition in price, has, we fear, become in many cases a down grade competition in the quality and character of the medical attendance supplied. That this competition has already touched questions of morality is shwon by the readiness with which contract work is accepted for fees which could not "pay" if the sort of attendance given were equal to what is given and expected in the case of attendance upon ordinary private patients of the same class, and so long as this sort of thing goes on, however agreeable to one's self-complacency it may be to talk about high aims and to lay down ethical rules as to our conduct one to another, there must be an air of unreality about the whole discussion. It would seem a reasonable thing and a fair basis on which the whole profession might unite—if they can unite on anything—to demand that a payment of one penny a week should be taken as the minimum fee for contract medical service. If one could put ethics on one side for a little while, talk less about high aims, but act loyally to one another on this one point, and combine to demand this wretched penny a week for contract work, we should see some hope of organisation for better things. But if we cannot unite even to prevent professional reward sinking to the miserable depths to which it is being dragged by the institution of juvenile clubs and other inventions of the middleman, depths at which no one can believe it possible to give good service and do honest work, then we may as well give up our dreams.

### Personal Infection in Typhoid Fever.

WE have several times lately had occasion to urge the more general recognition of the fact that, true as it is that in great epidemics of typhoid fever the infection is usually distributed either by milk or by water, it is not true, as is so commonly taught, that there is no danger of personal infection from case to case among people dwelling together as people do dwell together in the ordinary circumstances of life. A recent report by Dr. Priestley, medical officer of health for Lambeth, thoroughly bears out the importance of personal infection as one of the modes by which the disease is spread. In three localised outbreaks he traced out the apparent relation between the cases, and while failing to find anything to indicate that the disease had been spread by milk or water or shell fish, he was able to find histories of intercommunication between the individuals of each group such as would explain its distribution by direct contact or by indirect infection through clothes, food, &c. If all sick people and those attending on them were clean both in person and in habits, or if typhoid fever declared its nature from the beginning, so that in the management of it proper precautions might be taken, personal infection would not be a matter of frequency or of importance. Unfortunately typhoid fever has often run through a not inconsiderable portion of its course before it is satisfactorily diagnosed, and thus it happens, especially in the homes of those who are not well off, that many risks are run before the possibility of infection is considered. Just as in regard to water it is the fouling of the supply that is the danger, so in regard to the domestic spread of infection it is the soiled linen, the soiled food, the soiled person, that become the vehicles by which the disease is spread. We are afraid that to suggest that all people should live cleanly lives is a mere counsel of perfection, hence the supreme importance of early diagnosis of this treacherous disease. Sanitary authorities who provide free facilities for the bacteriological diagnosis of doubtful cases deserve every credit for their enlightened action.

### Our Undermanned Army Medical Service.

WHAT we are now learning in regard to the evils connected with an undermanned army medical service our friends in the United States have already learned from hard experience in Cuba, and Bills are to be introduced into Congress during the present session with the object not only of improving the efficiency of the various branches of their army medical department, but more especially of increasing the numbei of the commissioned medical officers on the peace establishment. The United States had 192 commissioned medical officers, and when war broke out these had immediately to be supplemented by many hundreds of untrained civilians. The result was, for a time at least, disaster and a large loss of life. In our own case, even during peace we have long been short of medical officers. For years past the cry has gone up that surgeons were so scarce that they had to be denied their proper leave—a deprivation which has tended greatly to undermine the popularity of the service. Was it, then, to be wondered at that, when war came upon us, the equipment cf the first few divisions used up all the available medical officers, and that almost from the outset civilian aid has had to be relied upon? The harsh teachings of experience have, however, impressed upon our American cousins the fact that the army medical officer is, or ought to be, a highly-trained specialist, who cannot be replaced offhand by any civilian, however skilful in his own line of practice. He is something more than a mere medical practitioner even in times of peace, and in war his duties are quite different—special and distinct. He may not be so good an operator as the civilian fresh from a great hospital, but, per contra, the trained operator may be even more at sea in regard to the daily duties of the military surgeon. The truth has dawned upon the Americans, as we hope it will soon dawn upon our own authorities, that an Army Medical Corps cannot be improvised any more than a Corps of Engineers, and that, as is recognised in regard to the latter, if efficiency in time of war is to be ensured, a full complement must be maintained even in time of peace.

